# School children brief training to save foreign body airway obstruction

**DOI:** 10.1007/s00431-023-05202-x

**Published:** 2023-09-30

**Authors:** Santiago Martínez-Isasi, Aida Carballo-Fazanes, Cristina Jorge-Soto, Martín Otero-Agra, Felipe Fernández-Méndez, Roberto Barcala-Furelos, Verónica Izquierdo, María García-Martínez, Antonio Rodríguez-Núñez

**Affiliations:** 1 CLINURSID Research Group, Psychiatry, Radiology, Public Health, Nursing and Medicine Department, Universidade de Santiago de Compostela, Santiago de Compostela, Spain; 2 Simulation, Life Support, and Intensive Care Research Unit, (SICRUS) of the Health Research Institute of Santiago de Compostela (IDIS), Santiago de Compostela, Spain; 3 Faculty of Nursing, Universidade de Santiago de Compostela, Av/Xoan XXIII, S/N, 15782 Santiago de Compostela, Spain; 4 Primary Care Interventions to Prevent Maternal and Child Chronic Diseases of Perinatal and Developmental Origin (RICORS), Instituto de Salud Carlos III, RD21/0012/0025 Madrid, Spain; 5 REMOSS Research Group, Faculty of Education and Sport Sciences, Universidade de Vigo, Pontevedra, Spain; 6 Escuela de Enfermería de Pontevedra, Universidade de Vigo, Pontevedra, Spain; 7 Lucus Augusti Universitary Hospital, Lugo, Spain; 8 Pediatric Critical, Intermediate and Palliative Care Section, Pediatric Area, Santiago de Compostela’s University Clinic Hospital, Santiago de Compostela, Spain

**Keywords:** Airway obstruction, Training, Kids save Lives, Teachers, School, Basic life support

## Abstract

Foreign body airway obstruction (FBAO) is a relatively common emergency and a potential cause of sudden death both in children and older people; bystander immediate action will determine the victim’s outcome. Although many school children’s basic life support (BLS) training programs have been implemented in recent years, references to specific training on FBAO are lacking. Therefore, the aim was to assess FBAO-solving knowledge acquisition in 10–13-year-old school children. A quasi-experimental non-controlled simulation study was carried out on 564 ten-to-thirteen-year-old children from 5 schools in Galicia (Spain). Participants received a 60-min training led by their physical education teachers (5 min theory, 15 min demonstration by the teacher, and 30 min hands-on training) on how to help to solve an FBAO event. After the training session, the school children’s skills were assessed in a standardized adult’s progressive FBAO simulation scenario. The assessment was carried out by proficient researchers utilizing a comprehensive checklist specifically designed to address the variables involved in resolving a FBAO event according with current international guidelines. The assessment of school children’s acquired knowledge during the simulated mild FBAO revealed that 62.2% of participants successfully identified the event and promptly encouraged the simulated patient to cough actively. When the obstruction progressed, its severity was recognized by 86.2% and back blows were administered, followed by abdominal thrusts by 90.4%. When the simulated victim became unconscious, 77.1% of children identified the situation and immediately called the emergency medical service and 81.1% initiated chest compressions. No significant differences in performance were detected according to participants’ age.

*  Conclusion*: A brief focused training contributes to prepare 10–13-year-old school children to perform the recommended FBAO steps in a standardized simulated patient. We consider that FBAO should be included in BLS training programs for school children.

**Table:** 

**What is Known:** *• Kids Save Lives strategy states that school children should learn basic life support (BLS) skills because of their potential role as first responders.* *• This BLS training does not include content for resolving a foreign body airway obstruction (FBAO).*	
**What is New:** *• Following a 60-min theoretical-practical training led by physical education teachers, 10–13-year-old school children are able to solve a simulated FBAO situation.* *• The inclusion of FBAO content in BLS training in schools should be considered.*

**Supplementary Information:**

The online version contains supplementary material available at 10.1007/s00431-023-05202-x.

## Introduction

Foreign body airway obstruction (FBAO) is a relatively common emergency and a potential cause of sudden death both in children and older people [1–4]. The outcome of an FBAO event depends to a large extent on the early removal actions; therefore, bystanders play a critical role in helping victims save their lives [5].

Considering children’s learning ability and their potential role as first responders, basic life support (BLS) training has been promoted and implemented in schools in different countries over the last decade [6], in part following the “Kids Save Lives” (KSL) initiative [7, 8]. The KSL statement, supported by the European Resuscitation Council (ERC) and the World Heart Organization, focuses on the training of school children (aged 12 years and younger) in cardiopulmonary resuscitation (CPR) as potential bystanders and even as trainers of others [7, 8]. In this regard, current evidences have shown that training school children in BLS is an effective and sustainable way to increase bystander efforts, and even survival after out-of-hospital cardiac arrest could be increased up to threefold [9–11].

However, KSL does not include reference to FBAO immediate management, perhaps due to the relative lack of evidence on FBAO training in school children [12]. In this sense, some teachers have reported that they usually focus of BLS training on the emergency call and CPR to a greater extent than on FBAO [13].

Despite prior experiences about FBAO management by laypeople [14, 15], children [16, 17] and school teachers [18], to the best of our knowledge, studies that assessed the skills acquisition by school children are lacking.

We conducted a quasi-experimental simulation study with 10–13-year-old students to test our hypothesis that a simple and brief simulation-based training, led by physical education (PE) teachers in schools, would improve the ability of school children to manage FBAO.

## Materials and methods

### Design

A quasi-experimental (non-randomized) simulation study design was performed using a non-controlled design.

### Participants

#### Schools

The recruitment process for the convenience schools (selected based on the number of pupils and their availability to facilitate and collaborate in the study) began by reaching out to the head teacher to provide him/her with the detailed information about the project’s methodology, and to obtain school-granted permission to be included in the study.

#### School children

Initially, 564 children aged 10 to 13 years old and attending to 5 public charter schools in Galicia (Spain), during the academic year 2021–2022, were screened. According to our country’s (Spain) education organization, the school children’s age corresponded to the last year of primary education (10–11 years old) and the first 2 years of high school (11–12 and 12–13 years old).

#### Inclusion criteria

We required participants to be between 10 and 13 years of age, to be currently enrolled in one of the participating schools, and to be naïve about BLS and FBAO management.

#### Exclusion criteria

Although they were included in the training activities, school children with any psychological or physical disability that would hinder their ability to perform the study procedures were excluded from the study assessments. Additionally, individuals were excluded from the assessment and data analysis if they missed any of the scheduled training or assessment sessions.

All children meeting the inclusion criteria who assented and had obtained written informed consent from their parents were finally included in the research (for both training and systematic assessment). In addition, just before commencing the study, verbal consent was obtained from all participating children. The research was approved by the Ethics Committee of the Faculty of Education and Sport Sciences – University of Vigo (Spain) (Code: 09–170123).

### Assessors and raters

The main investigator (also project coordinator) trained the research team for both the training and assessment methods. All the team members were healthcare personnel (physicians or nurses) with prior experience in BLS training and conducting performance BLS assessments for research studies. Each evaluation was carried out by a research team comprising a minimum of 6 evaluators and a general coordinator. A total of 18 evaluators participated in the assessment process.

The assessors’ and raters’ training consisted of an initial session outlining the project’s objectives, methods, and goals. This session specifically covered the training and assessment methodology, with the aim to minimizing potential biases. Before each evaluation session, a briefing was conducted to review the evaluations for that day, recall important points, and organize the assessors. In addition, a brief post-evaluation debriefing was conducted, at the end of the session.

### Intervention

**Fig. 1:**
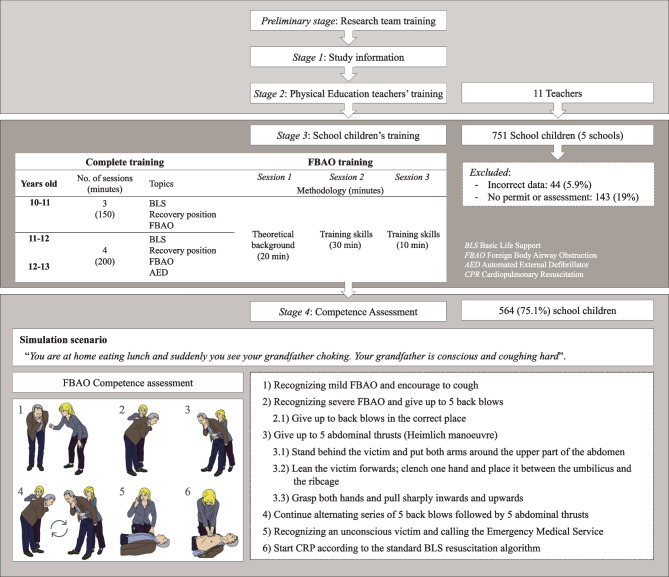
Flowchart study design

The study consisted of the following four stages (Fig. 1).

- *Stage 1—Study general information*: Information meetings were organized with the management teams of the five participating schools and the children’s legal guardians, in which the objectives, design, and detailed methods of the study were explained and the necessary permissions to take part were requested.
- *Stage 2—Physical education teachers’ training*: A total of 11 teachers were responsible for training the participating children at the school where they taught. All involved PE teachers underwent and passed a theoretical and practical provider- and instructor-dedicated course conducted by the research team (two ERC BLS nurse instructors and the project coordinator).
- The theoretical background (10 h; distance learning) sessions were delivered through the open-source learning platform *Moodle*. Teachers watched videos and completed learning assessment tests available on *Moodle* for 1 month. The practical hands-on training (lasting 2 h; face-to-face learning) was conducted by two instructor nurses. In this session, PE teachers were involved in standardized FBAO simulation scenarios. In addition, the project coordinator emphasized the key aspects of school children pedagogics and provided a methodological guide to try to standardize the messages that the teachers would give to the participant school children.
- *Stage 3—School children’s training*: PE teachers were responsible for children’s training with a maximum pupil/teacher ratio of 1:25 for 10–11-year-olds and 2:30 for 11–12- and 12–13-year-olds. The training content was standardized by following a guide with the content, methodology, structure, and timing of the sessions so that all pupils received the same information and have the opportunity perform the same hands-on training. Content included the BLS sequence, placement of the victim on the recovery position, FBAO immediate management, and automated external defibrillator (AED) use. The didactic methodology consisted of the sequence Explain-Demonstrate-Practice. Training sessions’ duration was adjusted according to each content (Fig. 1), but at least 60 min (20 theoretical and 40 practical) was devoted to each block.
- The specific FBAO training, which followed the protocol currently recommended in the ERC guidelines [20], was divided into three sessions (Fig. 1). The first one was focused on the theoretical background of FBAO immediate management by a layperson (20 min, 5 min of theory and 15 min of teacher’s demonstration), and the second and third sessions on the practical training of the skills needed to try to solve an initially mild that progressed to severe FBAO event. The practical hands-on session lasted 30 min (20 and 10 min in the second and third sessions, respectively). In the second session, the school children trained in pairs to practice the skills and, in the third one, they practiced a complete simulated FBAO scenario (first mild obstruction turning into severe obstruction).
- *Stage 4—Competence (outcome) assessment*: Each participant child was assessed by means of a standardized adult FBAO simulation scenario that took place between 1 and 15 days after the training day. In the assessment scenario, the child was alone and was told that “*You are at home eating and suddenly you notice your grandfather choking. Your grandfather is conscious and coughing hard*.” A standardized patient (small adult), trained beforehand, pretended to be the victim and acted on precise cues that simulated a mild obstruction, evolving into a severe FBAO and, subsequently, an unconscious victim. The case ended when the child expressed their intention to perform chest compressions on the unconscious victim. Anthropometric (body weight and height) and demographic (gender and date of birth) variables of the participants were also collected.

### Competence (outcome) assessment details

The current recommended protocol [20] for treating an FBAO adult event was used as the basis for the assessment tool. The steps outlined in the guidelines (1 to 6) can be seen in the “FBAO competence assessment” section of Fig. 1. The assessment was conducted by the research team (with one rater per child), previously trained and using a checklist (according to the “FBAO competence assessment” variables shown in Fig. 1) that evaluated not only the initiation and completion of each step but also the accuracy of its execution.

### Statistical analysis

All analyses were performed with IBM SPSS Statistics version 20.0 for Windows. The Kolmogorov–Smirnov test was used to analyze data distribution and normality. The results of quantitative variables (weight and height) were expressed as median (interquartile range) and qualitative variables (FBAO sequence) as absolute frequencies (relative frequencies), as appropriate. Between-group comparisons used *χ*^2^ and Fisher’s exact tests. A *p*-value less than 0.05 was considered statistically significant.

## Results

The total number of school children trained and invited to participate in this study was 751, and after excluding 187 (24.9%) because of incorrect or incomplete data, finally, 564 school children comprised the final sample included in the analysis (55% girls). The participants were in one of three age groups (according to school stage): 10–11 years old (34.8%), 11–12 years old (32.6%), and 12–13 years old (32.6%), and their characteristics are shown in Supplementary Material 1.

Table 1 shows the school children who correctly performed each of the recommended steps for solving an FBAO. Coughing was identified by 62.6% of participants as a sign of mild FBAO, and consequently, they encouraged to cough and 86.2% were able to detect severe FBAO by administering back blows. Abdominal thrusts (*Heimlich* maneuver) were administered by 90.4% of the school children when the FBAO did not solve with back blows. However, only half (52%) of the participants alternated the 5 back blows and 5 abdominal thrusts continuously after the first Heimlich maneuver attempt.

When the victim simulated unconsciousness, 77.1% of the participants called EMS and 81.8% started chest compressions.

**Table 1:** Step-by-step FBAO performance of total participants and by age group

	**All**	**10–11 years**	**11–12 years**	**12–13 years**	***p*-value**
Recognizing mild FBAO and encouraging to cough	351 (62.6)	118 (60.5)	107 (58.8)	126 (68.5)	0.122
Recognizing severe FBAO and give up to 5 back blows	486 (86.2)	166 (84.7)	159 (86.4)	161 (87.5)	0.726
Give up to back blows in the correct place	403 (82.9)	137 (82.5)	130 (81.8)	136 (84.5)	0.802
Give up to 5 abdominal thrusts (*Heimlich* maneuver)	510 (90.4)	179 (91.3)	158 (85.9)	173 (94.0)	**0.025**
Stand behind the victim and put both arms around the upper part of the abdomen	496 (97.3)	176 (98.3)	152 (96.2)	168 (97.1)	0.488
Lean the victim forwards; clench one hand and place it between the umbilicus and the ribcage	388 (78.2)	142 (80.7)	117 (77.0)	129 (76.8)	0.616
Grasp both hands and pull sharply inwards and upwards	359 (92.8)	130 (91.5)	112 (96.6)	117 (90.7)	0.164
Continue alternating series of 5 back blows followed by 5 abdominal thrusts	293 (52.0)	102 (52.0)	97 (52.7)	94 (51.4)	0.967
Recognizing unconsciousness and calling EMS	431 (77.1)	161 (83.0)	132 (72.5)	138 (75.4)	**0.044**
Start CPR	454 (81.1)	158 (80.6)	153 (83.2)	143 (79.4)	0.652

Significant values are in bold

Inter-group analysis with the chi-square test

Categorical variables expressed as absolute frequency (relative frequency)

*FBAO* foreign body airway obstruction, *EMS* emergency medical service, *CPR* cardiopulmonary resuscitation

When the victim simulated unconsciousness, 77.1% of the participants called EMS and 81.8% started chest compressions.

**Fig. 2:**
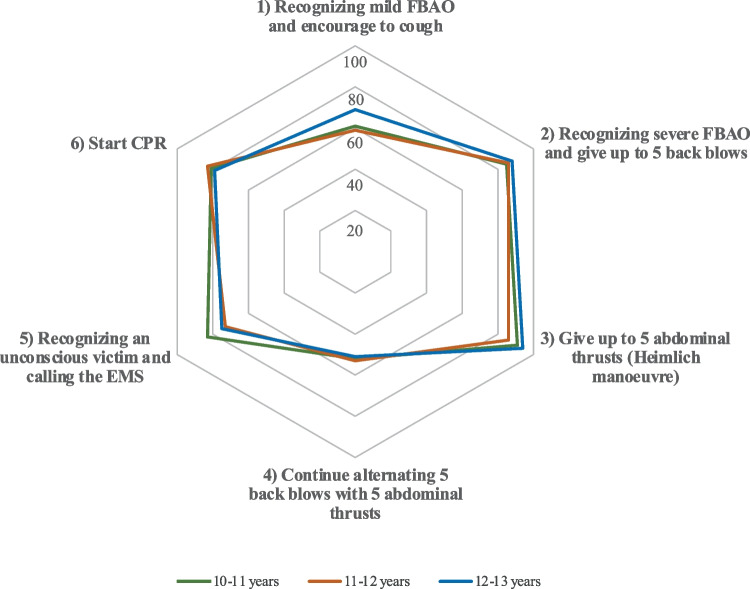
Performance of the recommended steps for the treatment of the adult victim with FBAO by age group. Hexagonal concentric lines from 20 to 100 represent the percentage of school children performing each step

The spider graph of Fig. 2 shows the percentage of school children by age group who were able to perform the steps of the protocol adequately. The only step that was performed by less than 60% of participants in all groups was the continuation of series of 5 back blows and 5 abdominal thrusts.

Significant differences between groups (Table 1) were found only in the administration of abdominal thrusts (*p* = 0.025) finding a pairwise difference between the 10–11-year-old group (performed by 91.3% of school children) and the 11–12-year-old group (85.9%; *p* = 0.007); and EMS call (*p* = 0.044) between the 10–11-year-old group (carried out by 83.0% of participants) with the 11–12-year-old group (72.5%; *p* = 0.010); and the 10–11-year-old group with 12–13-year-old group (performed by 72.5% vs 75.3% of participants respectively; *p* = 0.046).

## Discussion

The cornerstone of the FBAO management is to eliminate the cause of the obstruction as soon as possible, being the laypeople who witness the event key element in its resolution that will determine the victim’s prognosis. KSL initiative [7, 21] promotes children as the ideal target population to be trained as first responders considering the essential role of bystanders in the chain of survival. In this sense, our study has shown how 10–13-year-old school children are able to learn how to manage a simulated FBAO event after a brief training program led by their PE teachers. We consider our results relevant as no evidence of similar studies has been found and they could provide a basis for implementing FBAO management training in schools.

We observed that after the training, at least 75% of the participants were able to perform almost all the steps of the FBAO protocol properly. However, the school children were slightly less proficient to recognize mild FBAO and encouraging cough and continuing alternating series of 5 back blows followed by 5 abdominal thrusts when indicated (severe obstruction and failure of first attempt). These results are consistent with previous studies [22, 23] performed with adults and health science students, respectively, in which these two steps of the protocol were also the least performed, being carried out by less than half of the participants. Although it would be convenient to corroborate these findings with additional studies, we consider that it will be necessary to reinforce these two messages during laypeople training.

Nevertheless, abdominal thrusts were performed by more than 90% of school children in our study, which also is in line with the data reported by Carballo-Fazanes et al. [22] and Cardalda-Serantes et al. [23]. We hypothesize that this could be due to “indirect” knowledge, since airway clearance maneuvers are often limited to the “Heimlich maneuver,” for instance in movies.

Concerning BLS training, the gradual implementation of competencies and contents in the school curriculum adapted to the different ages of children, starting from the basics and adding increasingly complex elements, has been considered a reference [11, 19]. The participants in our study have received all the training related to BLS, recovery position, FBAO, and AED together. Future studies should test whether sequential training from the early years leads to better retention of FBAO content in later years.

In this sense, the age of children has been considered a limiting factor for some techniques, for example, to reach the adequate chest compressions depth suggesting 12 years as the age from which acceptable quality can be achieved [7, 24]. However, it has also been pointed out that the anthropometric characteristics of the rescuer are more influential than age in itself and, therefore, what will condition the performance of certain techniques [25, 26]. This could be extrapolated to the performance of back blows and abdominal thrusts in the case of FBAO, considering the execution of these techniques to be more complex and ineffective when done by younger rescuers.

However, the ideal height or strength required to effectively perform the Heimlich maneuver or back blows is unknown because, in addition to the anthropometric characteristics of the rescuer (not only age), it will also depend on the anthropometry of the victim. Nevertheless, this training not only benefits school children in handling situations involving older victims, but also equips them with skills to address effectively with FBAO events that may happen to peers or siblings of similar anthropometric dimensions. De Buck et al. [12] suggested that children aged 11–12 should be aware of the differences between mild and severe FBAO and administer first aid in case of choking by correctly applying back blows. They also stated that 13–14-year-olds should also be able to perform abdominal thrusts. In this line, the official school curriculum in Spain [27] establishes that action in an FBAO situation should be trained from the age of 13–14 years. Nevertheless, the curriculum only provides for learning the “Heimlich maneuver,” incorrectly reducing, as mentioned above, the FABO protocol to a single step.

Based on our results, it seems reasonable to include FBAO training in the school curriculum at the age of 10–11 years, as no significant differences were observed between this age group and older children. On the other hand, considering that younger children have been shown to be able to learn the main components of BLS [28, 29], future studies could consider the inclusion of FBAO training content before the age of 10–11 years. Based on this previous evidence, even if they do not have the anthropometric conditions to perform the technique with quality and effectiveness, knowing the protocol could help them to identify the emergency situation and alert EMS, and could also facilitate future learning.

Other further key elements in the BLS training of school children are related to the trainers and training methodology. In this sense, in our study the school children were trained by their PE teachers during school hours, using 60 min (20 min of theory and demonstration by the teacher and 40 min of supervised practice). Our decision was based on previous evidence which has highlighted the ability of trained teachers to effectively teach BLS to children [7, 30, 31] which, together with their pedagogical skills in adapting the training to the different ages and characteristics of their pupils [24, 28], may make them the most suitable instructors [19].

In this connection, it was observed after the training that more than 75% of the participants successfully completed almost all the steps of the FBAO protocol. Although no evidence exists about which educational strategy to teach school children is the most effective [19, 32], our data point that the methodology used, focusing mainly on skills, as well as the time devoted to training is reasonable to achieve the proposed goals and is in line with previous studies on school children BLS [33, 34] and AED [34, 35].

In view of the positive results of our study added to the willingness of teachers to provide BLS training shown in previous studies [13, 36], it seems reasonable to include the FBAO bystander management in the school children curriculum, following KSL strategy.

### Limitations

This study was carried out in a specific geographical and cultural area and school children were all enrolled in public charter schools, which hampers the generalizability of the results. On the other hand, the FBAO simulated scenario does not reproduce a real-life situation where stress and a different environment at school could affect the children’s performance. In addition, retention over time of knowledge and skills was not explored in our study. Finally, the training of the children was conducted by 11 different teachers. While it cannot be guaranteed that the transmitted training was identical in all cases, emphasis was placed on the importance of consistent training with the standardized content during teacher training sessions. Moreover, owing to the extensive participation of multiple teachers and schools, the evaluation phase extended from 1 to 15 days after the training period. Consequently, potential variations in knowledge retention could have arisen between children assessed on the initial day versus those evaluated on day 15. However, it is essential to note that the time frame was deemed insufficient for any significant retention differences to emerge [7, 37].

## Conclusions

A brief theoretical-practical training (60 min) led by PE teachers contributes to prepare 10–13-year-old school children to perform the recommended steps for treating FBAO in a simulated adult choking scenario. As the step referring to the continuation of 5 back blows followed by 5 abdominal thrusts after failing a first attempt was the weakest performed, in future training sessions, special attention should be paid to this step of the protocol. We consider that our results should encourage the incorporation of FBAO immediate management training in schools.

### Supplementary Information

Below is the link to the electronic supplementary material.

Supplementary file1 (DOCX 15 KB)

## Data Availability

All relevant data can be found in the manuscript and in the supplementary information files. For further information, please contact the corresponding author.

## Abbreviations

AED: Automated external defibrillator
BLS: Basic life support
CPR: Cardiopulmonary resuscitation
EMS: Emergency medical service
ERC: European Resuscitation Council
KSL: Kids Save Lives
FBAO: Foreign body airway obstruction
PE: Physical education
