# Supplementary material for: School children brief training to save foreign body airway obstruction
Source: Eur J Pediatr. 2023 Sep 30;182(12):5483–91. doi: 10.1007/s00431-023-05202-x (PMC10746610; doi:10.1007/s00431-023-05202-x)
Supplement: Supplementary file 1 — Supplementary file1 (DOCX 15 KB) [file 431_2023_5202_MOESM1_ESM.docx]

| **Supplementary Table 1**. Participants’ characteristics | | | |
| --- | --- | --- | --- |
| Weight _(kg)_ | | | 48.9 (42.5-56.6) |
| Height _(cm)_ | | | 157.4 (151.6-163.1) |
| Gender | | Male | 253 (44.9) |
|  |  | Female | 311 (55.1) |
| Years-old | | | |
| 10-11 | Year of birth | | 2011 |
|  | Weight _(kg)_ | | 43.7 (39.2-51.6) |
|  | Height _(cm)_ | | 152.1 (148.0-157.0) |
|  | Gender | Male | 94 (48.0) |
|  |  | Female | 102 (52.0) |
| 11-12 | Year of birth | | 2010 |
|  | Weight _(kg)_ | | 49.8 (43.2-56.4) |
|  | Height _(cm)_ | | 157.3 (153.0-163.0) |
|  | Gender | Male | 79 (42.9) |
|  |  | Female | 105 (57.1) |
| 12-13 | Year of birth | | 2009 |
|  | Weight _(kg)_ | | 53.2 (47.0-60.3) |
|  | Height _(cm)_ | | 162.8(158.2-168.6) |
|  | Gender | Male | 80 (43.5) |
|  |  | Female | 104 (56.5) |
| Continuous variables expressed as median (interquartile range)  Categorical variables expressed as absolute frequency (relative frequency). | | | |
